# Current trends in the medical treatment of neuropathic low back pain: a Swedish registry-based study of 1.7 million people

**DOI:** 10.1186/s12891-024-07599-4

**Published:** 2024-06-20

**Authors:** Linus Nyqvist, Josefin Åkerstedt, Olof Thoreson

**Affiliations:** 1 Spine Unit, Department of Orthopedics, Umeå University Hospital, Umeå, Sweden; 2 Department of Diagnostics and Intervention, Orthopedics and Spine Surgery, Umeå University, Umeå, Sweden; 3 Department of Orthopedics, Institute of Clinical Sciences at Sahlgrenska Academy, University of Gothenburg, Gothenburg, Sweden; 4 Research and Development Primary Health Care Center, Gothenburg, Sweden

**Keywords:** Neuropathic low back pain, Radicular pain, Opioids, Neuropathic analgesics, Pain therapy

## Abstract

**Background:**

Low back pain, a common problem worldwide, causes more global disability than any other condition and is associated with high costs to society. This observational registry-based study describes the current trends in the medical treatment of neuropathic low back pain in the Swedish region of Västra Götaland, which has a population of 1.7 million. The study aims to; (1) identify the prevalence of neuropathic low back pain within the study population; (2) to explore the patterns of medical treatment utilization, including the prevalence and distribution of opioids (OG) and analgesics specified for neuropathic low back pain (NG) and (3) to evaluate the long-term trends and changes in medical treatment practice for neuropathic low back pain over the study period.

**Methods:**

This study includes a descriptive analysis of aggregated data extracted from the Swedish primary care registry VEGA and the pharmaceutical prescription registry Digitalis between the years 2017 and 2021. The data were stratified by year, age, gender, pharmaceutical code (ATC), and sub-diagnoses and presented as the prevalence of unique patients retrieving prescribed medication within six months before or after a registered diagnosis of neuropathic low back pain. The pharmaceutical codes were furthermore grouped into two groups depending on their mechanism of action; opioid group (OG) and neuropathic group (NG).

**Results:**

In all four diagnosis groups, more patients used opioid analgesics than neuropathic analgesics. The greatest difference between the opioid group and neuropathic group was in the lumbar spinal stenosis diagnosis group (67.1% vs. 40.6%), followed by the lumbar root canal stenosis diagnosis (65.9% vs. 44.2%), the nerve root and plexus compressions in intervertebral disc disorders diagnosis (57.5% vs. 40.8%), and lumbago with sciatica diagnosis (38.4% vs. 22.7%).

**Conclusions:**

The trends suggest a general increase in the prescription rate and therefore patients’ use of neuropathic analgesics for neuropathic pain associated with the studied diagnoses. However, opioid treatment remains the most common. The results indicate that the treatment for neuropathic low back pain needs to be improved.

## Introduction

Low back pain is a common problem worldwide regardless of culture, geographical origin, or gender. The cause of low back pain might be multifactorial and is related to muscular, skeletal, and/or nerve-related problems. Low back pain is a major contributor to lost productivity and high costs for the health care system. Therefore, providing the best possible treatment and rehabilitation of these patient groups is important not only for the patient but also for society [1–6]. In Sweden, more than 600,000 people are diagnosed with symptomatic low back pain every year [7]. Only a few of these patients fulfills the diagnostic criteria to undergo surgical treatment, for many of the diagnoses in our study, the primary indication for surgery is pain and reduced quality of life. For those who are eligible for surgery, the majority (89%), according to the Swedish spine registry (Swespine), have taken analgesics intermittently or regularly before surgery. The same group indicates that they still have a lot of pain despite pain treatment (on average 7.2 out of 10 on the NRS-scale) [8], a finding that calls into question the current clinical medical treatment for low back pain.

Specific low back pain and nerve-affecting low back pain cause approximately 10% of all low back pain [9]. Lumbago with sciatica, lumbar spinal stenosis, lumbar root canal stenosis, and nerve root and plexus compressions in intervertebral disc disorders represent the largest diagnostic groups for neuropathic low back pain. The International Association for the Study of Pain (IASP) Special Interest Group on Neuropathic Pain (NeuPSIG) suggested in 2008 the definition of neuropathic pain as “pain arising as a direct consequence of a lesion or disease affecting the somatosensory system”. This is now a globally accepted definition. Neuropathic pain is often characterized by a burning sensation, stinging, or diffusely unpleasant pain and is often caused by compression or distraction that causes inflammation and irritation to the neural structures [10]. Most patients seeking care at onset of neuropathic low back pain are seen and diagnosed in the primary care [11–13].

Typically, the first line of treatment for low back pain includes non-prescriptive analgesics and physiotherapy. The medical treatment of low back pain must be adjusted according to the patient’s symptoms and pathology to achieve the best effect. Although the treatment protocols are developed regionally, treatment guidelines are similar throughout Sweden. The Västra Götaland region publishes an updated recommendation list (REK-list) every year. In 2022, the first recommended choice for medical treatment for peripheral neuropathic low back pain is amitriptyline, nortriptyline, gabapentin, or duloxetine; pregabalin is listed as the second recommended choice [14]. For neuropathic low back pain, analgesics such as paracetamol and NSAIDs have limited effects. For severe pain and acute pain, opioid treatment may be added. However, as opioids mainly target nociceptive pain, they seldom have a good effect on neuropathic pain [15].

The overall aim of this observational registry-based study is to comprehensively characterize the current trends in the medical treatment of neuropathic low back pain within the Swedish region of Västra Götaland, encompassing a population of 1.7 million. The study specifically seeks to achieve the objectives: [1] To identify the prevalence of neuropathic low back pain within the study population; [2] To explore the patterns of medical treatment utilization, including the prevalence and distribution of opioids (OG) and analgesics specified for neuropathic low back pain (NG) and [3] To evaluate the long-term trends and changes in medical treatment practice for neuropathic low back pain over the study period.

This study aims to provide valuable insights into the management and clinical outcomes of neuropathic low back pain in a large population register-based cohort. This information can inform healthcare providers, policymakers, and stakeholders in optimizing treatment strategies and improving patient care for individuals suffering from this common and crippling condition.

## Methods

This observational registry-based study describes the current trends in the medical treatment of neuropathic low back pain and evaluates differences over time (per year) for age, gender, pharmaceutical code (ATC), and sub-diagnoses and are presented as the prevalence of unique patients retrieving prescribed medication within six months before or after a registered diagnosis of neuropathic low back pain. These cohorts are compared to the general population.

The Swedish health care system is divided into primary care, referral required special care, and hospital/inpatient care. All diagnoses studied involve neuropathic low back pain and are most commonly diagnosed in primary care [16]. Sweden is divided into regions that provide and finance public health care. The target population of this study is the approximately 1.7 million inhabitants (1,744,859 inhabitants) of the Västra Götaland region. All the regions are reimbursed by the national government for primary health care based on factors such as diagnoses, patient visits, the number of patients listed, and number of patients treated [17]. This system encourages thorough diagnoses and diagnosis-based and symptom-based treatment. Treatment guidelines are similar throughout Sweden, although treatment protocols may be developed regionally.

### Definition of neuropathic pain

Neuropathic pain occurs due to a lesion somewhere in the somatosensory system. The definition of neuropathic pain as “pain arising as a direct consequence of a lesion or disease affecting the somatosensory system,” was suggested by the International Association for the Study of Pain (IASP) Special Interest Group on Neuropathic Pain (NeuPSIG) in 2008. The definition is globally accepted and applied in the region Västra Götalands Conventional analgesics usually have no or poor effect on neuropathic pain and is why the first-line preparations are tricyclics and/or antiepileptics.

### Data sources and research database

Aggregated data from 2017 to 2021 were extracted from the Swedish primary care registry VEGA and the medical prescription registry Digitalis for the Region of Västra Götaland. The Digitalis registry, introduced in 2013, includes all medications retrieved from outpatient pharmacies (i.e., prescriptions retrieved). The VEGA registry, introduced in 2014, includes all diagnoses registered by a clinic (primary, outpatient, and inpatient health care) funded by the public health care system (28,29). All opioids and analgesics for neuropathic pain included in this study are registered prescription drugs.

### Study sample

The study population includes all patients with registered diagnoses of lumbago with sciatica, nerve root and plexus compressions in intervertebral disc disorders, lumbar spinal stenosis, and lumbar root canal stenosis. The data were stratified by age, gender, year of diagnosis, and type of analgesic. Analgesics were separated into two groups: the neuropathic group (NG) and the opioid group (OG). The NG was prescribed amitriptyline, nortriptyline, gabapentin, or duloxetine. The OG were prescribed tapentadol, tramadol, codeine, combinations of codeine, buprenorphine, oxycodone, or combinations of oxycodone and morphine (Table 1).

**Table 1:** Diagnosis codes (ICD-10) and medication codes (ATC) of diagnoses and analgesics

Diagnoses	Opioid group (OG)	Neuropathic group (NG)
Lumbago with sciatica (M54.4)	Morphine (N02AA01)	Amitriptyline (N06AA09)
Nerve root and plexus compressions in intervertebral disc disorders (M51.1 K)	Buprenorphine (N02AE01)	Nortriptyline (N06AA10)
Lumbar spinal stenosis (M48.0 K)	Oxycodone (N02AA05)	Gabapentin (N03AX12)
Lumbar root canal stenosis (M48.8 K)	Oxycodone combinations (N02AA55)	Duloxetine (N06AX21)
	Tapentadol (N02AX06)	
	Codeine combined with paracetamol (N02AJ06)	
	Tramadol (N02AX02)	

Aggregated data were extracted and presented as quantity and proportion of unique individuals diagnosed with one or more of the included diagnoses who retrieved one or more of the included medications six months before or after the registered diagnosis. The study design allowed inclusion of patients who have transitioned between NG and OG analgesics during as well as those receiving both types of medications during the study period. Information about medication withdrawal in the opioid group and in the total population were also collected and used for comparative analysis.

### Statistical analysis

Descriptive analysis was the main analysis in this study. Statistics were processed in Microsoft Excel (version 16.66.1, Umeå, Sweden) and presented numerically. The data needed no further statistical analysis due to the complete coverage.

## Results

The results are based on the 1,744,859 inhabitants (2021) of the Västra Götaland region. The distribution between sexes were 50.4% men and 49.6% women. The patients were diagnosed between 2017 and 2021. The largest diagnosed group was the lumbago with sciatica group (M54) (*n* = 74,555). This group was followed by the patients diagnosed with nerve root and plexus compressions in intervertebral disc disorders (M51.1 K) (*n* = 16,041), the patients diagnosed with lumbar spinal stenosis (M48.0 K) (*n* = 7,678), and the patients diagnosed with lumbar root canal stenosis (M48.8 K l) (*n* = 2,013). All these groups include neuropathic low back pain symptoms.

For all these groups, there were more patients retrieving analgesics from the opioid group than the neuropathic group, both in number of individuals and percentage. The highest difference in percentage between the opioid group and neuropathic group was in the lumbar spinal stenosis diagnosis group (M48.0) (67.1% vs. 40.6%), followed by the lumbar root canal stenosis diagnosis (M48.8 K) (65.9% vs. 44.2%), the nerve root and plexus compressions in intervertebral disc disorders diagnosis (M51.1 K) (57.5% vs. 40.8%), and the lumbago with sciatica diagnosis (M54.4) (38.4% vs. 22.7%) (Table 2).

**Table 2:** Unique individuals who retrieved one or more analgesics six months before and after their registered diagnosis, between the years 2017–2021

	Number of individuals				Percentage				
Diagnosis code	Lumbago with sciatica	Nerve root and plexus compressions in intervertebral disc disorders	Lumbar spinal stenosis	Lumbar root canal stenosis		Lumbago with sciatica	Nerve root and plexus compressions in intervertebral disc disorders	Lumbar spinal stenosis	Lumbar root canal stenosis
**Neuropathic group (NG)**									
Men	6584	2 855	1 187	343		20.6	37.0	35.2	39.2
Women	10 348	3 682	1 933	546		24.3	44.2	44.9	48.0
**Total**	**16 932**	**6 537**	**3 120**	**889**		**22.7**	**40.8**	**40.6**	**44.2**
**Opioid group (OG)**									
Men	12 551	4 384	2 181	544		39.3	56.9	64.7	62.2
Women	16 044	4 840	2 972	782		37.6	58.1	69.0	68.7
**Total**	**28 595**	**9 224**	**5 153**	**1 326**		**38.4**	**57.5**	**67.1**	**65.9**

**Fig. 1:**
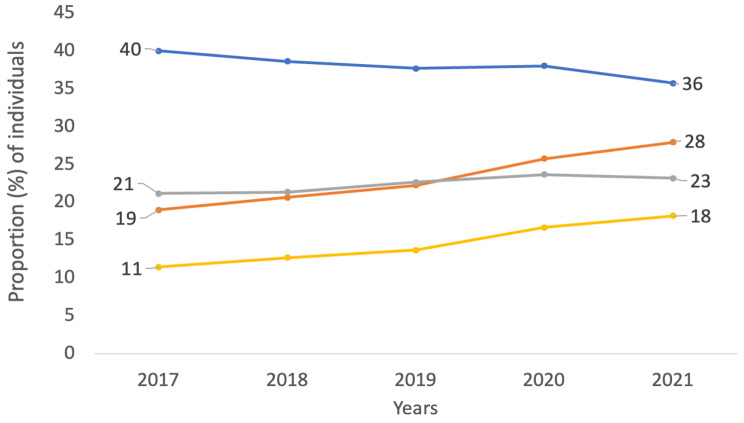
The proportion (%) of individuals per year with the diagnosis lumbago with sciatica (M54.4)who have collected one or more medications in the neurogenic (orange line) and opioid group (blue line) and the specific medications Gabapentin (yellow line) and Oxycodone (grey line) 6 months before or after registered diagnosis

**Fig. 2:**
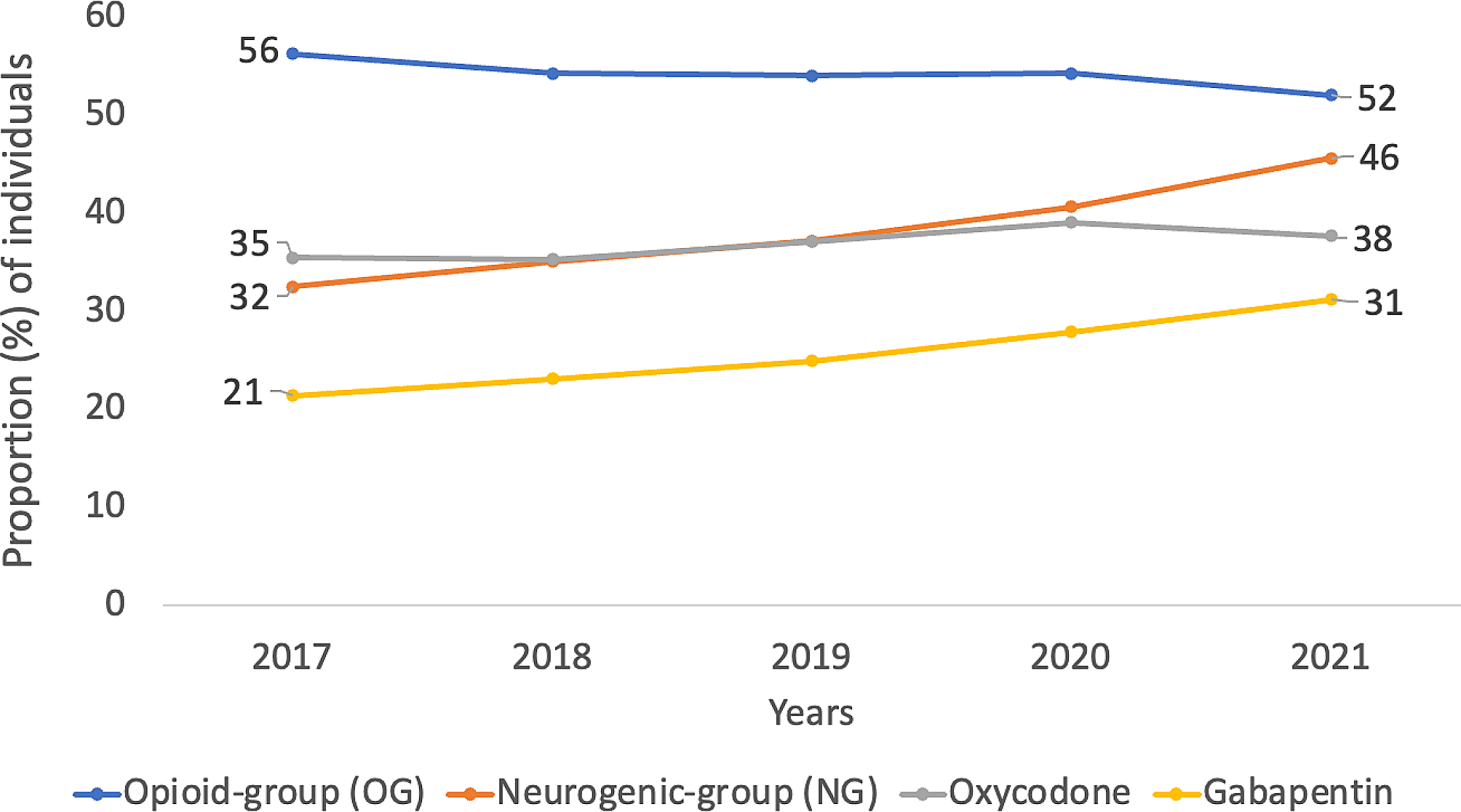
The proportion (%) of individuals per year *with diagnosis nerve root and plexus compression in intervertebral disc disorders (M51.1 K)*, who have collected one or more medic*ations from the neurogenic group (orange line), opioid group (blue line) and from the specific medications Gabapentin (yellow line) or Oxycodone (grey line)* 6 months before *or after* registered diagnosis

In the diagnosis group lumbago with sciatica (M54.4) and nerve root and plexus compression in intervertebral disc disorder (M51.1 K), which are the two largest diagnoses in terms of individuals in our study, the proportion of unique patients who retrieved analgesics from the neuropathic group showed a general increase between 2017 and 2021. However, opioid analgesics decreased for the same diagnosis M54.4 (Fig. 1) and M51.1 K (Fig. 2). The two analgesic groups prescribed oxycodone (OG) and gabapentin (NG) had the highest proportion of patients retrieving these drugs for all studied diagnoses. For these individual diagnoses, gabapentin retrieval increased, and oxycodone retrieval decreased. The highest proportion of patients retrieving oxycodone had a lumbar spinal stenosis diagnosis (53.4%), and the highest proportion of patients retrieving gabapentin had lumbar root canal stenosis diagnosis (31.7%).

**Fig. 3:**
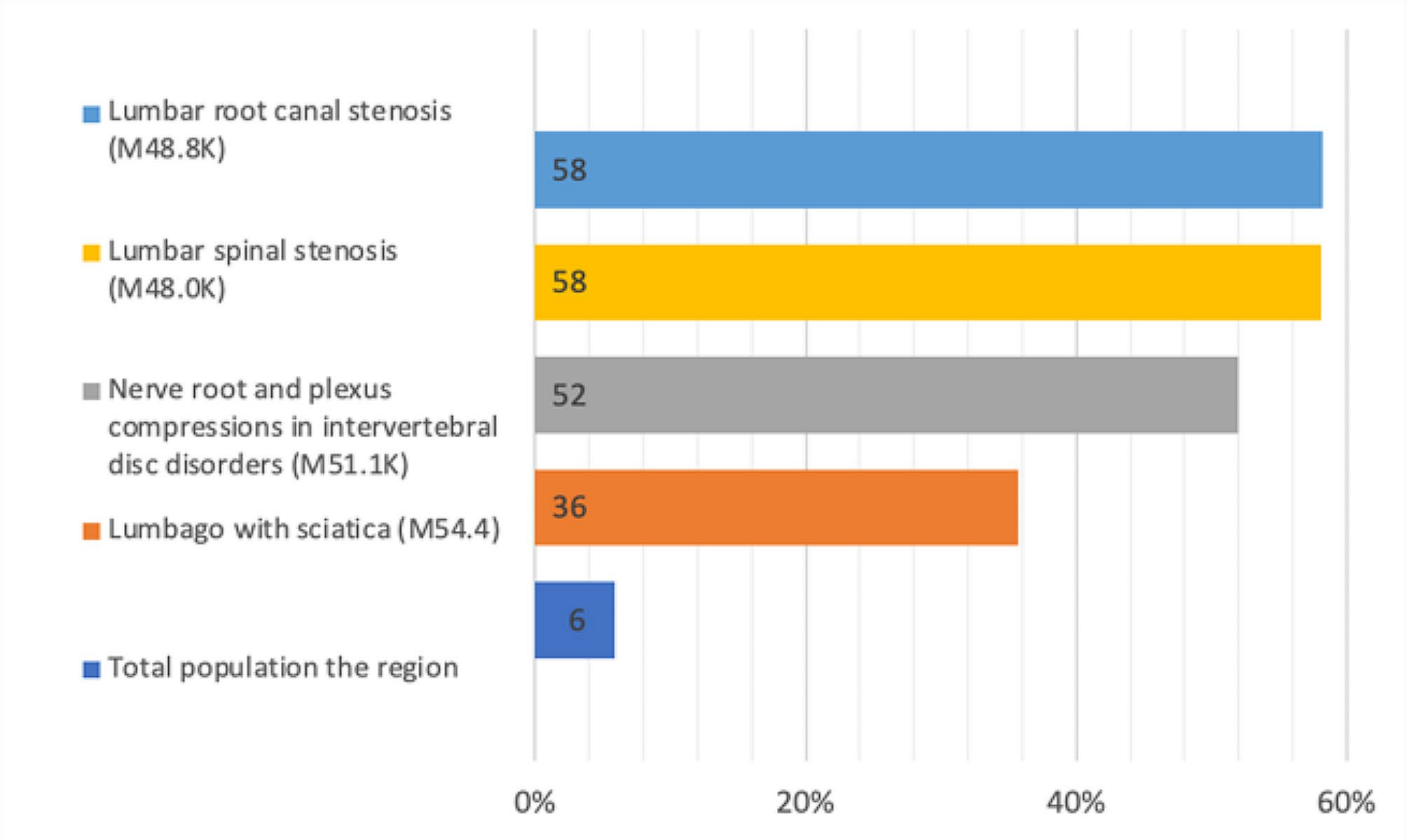
The proportion (%) of individuals retrieving opioid medications from the opioid group (OG) in 2021, subgrouped by diagnoses and compared to the total population in the region

**Fig. 4:**
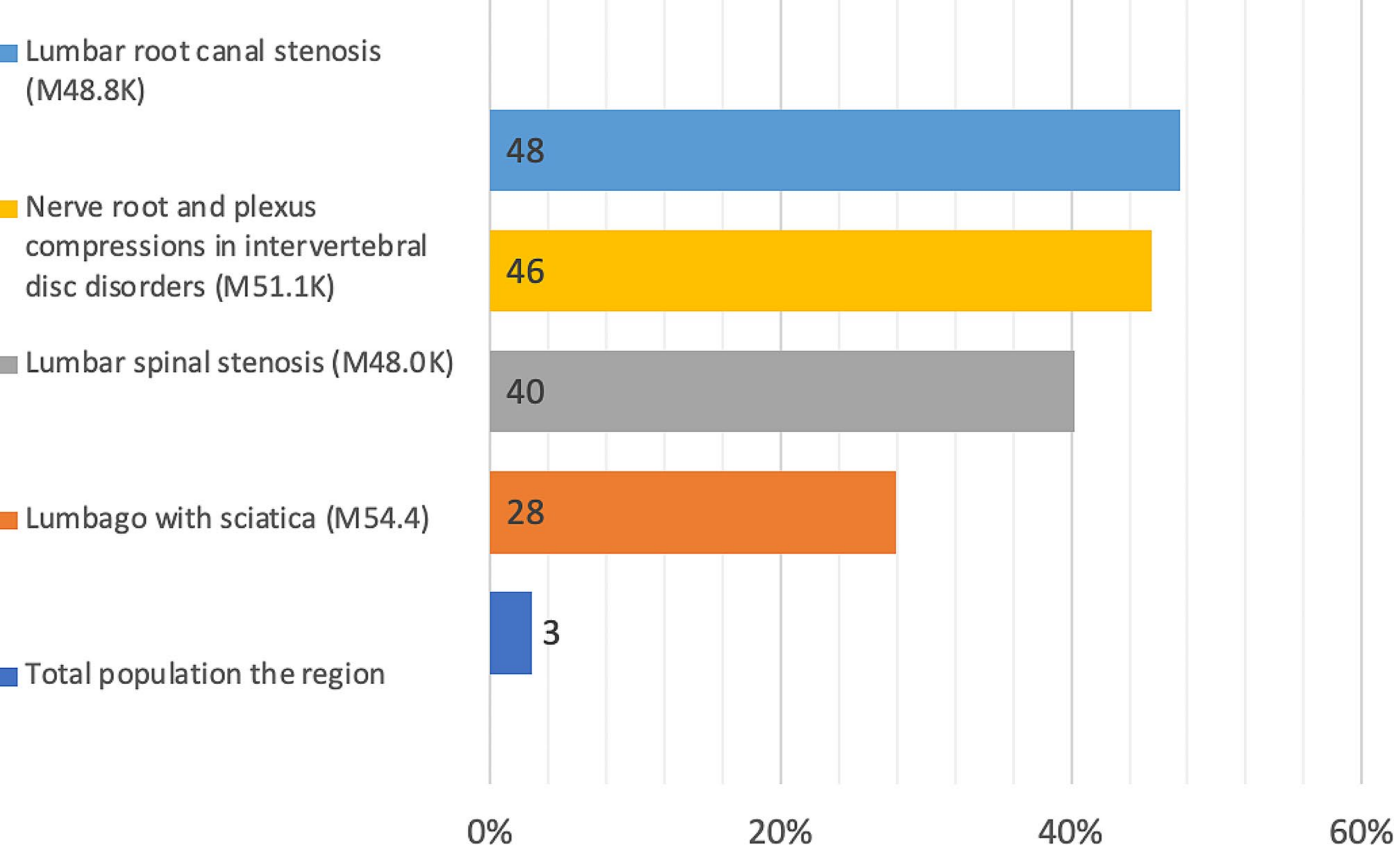
The proportion (%) of individuals retrieving neuropathic medications from the neuropathic group (NG) in 2021, subgrouped by diagnoses and compared to the total population in the region

**Fig. 5:**
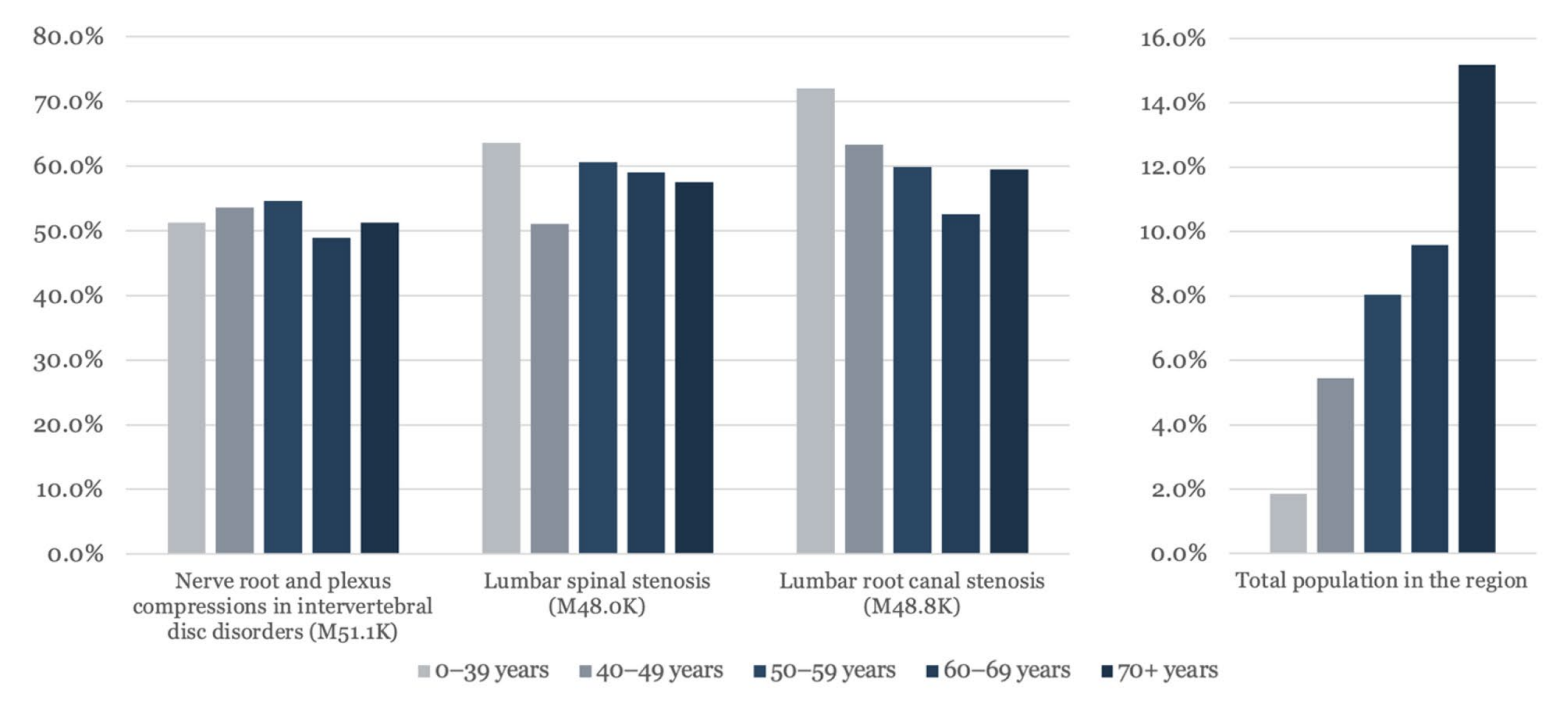
The proportion (%) of individuals retrieving opioids in 2021, subgrouped diagnosis and age compared to the retrieval of opioids in the total population during the same year

The trends in opioid retrieval were also studied and compared to those in the total population in 2021. This comparison was done to identify confounding diagnoses and why people were prescribed opioids. The results show a higher retrieval of opioids among our studied diagnoses compared to the total population; in some groups, this was almost ten times higher (Fig. 3). The same pattern is seen in the neuropathic medication *(NG-group)*, the retrieval is higher in our studied diagnoses compared to the total population (Fig. 4). The results were further stratified to age groups: 0–39; 40–49; 50–59; 60–69, and > 70 years. The results also show a trend of increased opioid retrieval in the total population in the higher age groups, but the distribution of opioid retrieval was evenly distributed in all age groups studied (Fig. 5).

The registry data have an even gender distribution (male 50.4% and female 49.6%). The only difference between the genders regarding the retrieval of analgesics was seen in the lumbar root canal stenosis group (M48.8 K) (48.0% vs. 39.2%). In proportion to all individuals within the studied diagnosis, the children (0–18 years old) accounted for 0.40% in lumbago with sciatica (M54.4), 0.37% in nerve root and plexus compression in intervertebral disc disorder (M51.1 K), 0.05% in lumbar spinal stenosis (M48.0) and 0.04% in lumbar root canal stenosis (M48.8 K).

## Discussion

Neuropathic low back pain is a challenging condition to treat effectively. Treatment starts with correct prescriptions from the physicians involved independent of treatment level (primary, outpatient, and inpatient health care).

In the Västra Götaland region, the proportion of patients retrieving neuropathic medications increased for the diagnosis groups lumbago with sciatica and nerve root and plexus compression in intervertebral disc disorder. These groups represent the two largest groups in the study. Lumbago with sciatica was the largest group as it is based on clinical findings alone; the other diagnosis includes radiological findings. The study does not account for the fact that patients may have more than one of the studied diagnoses and for patients with other diagnoses for which the same analgesics as those studied here are prescribed. It is also acknowledged that there is a complexity of chronic low back pain, which may involve nociceptive, nociplastic or mixed mechanisms of pain and that might be associated with and present in the patients with anyone of the studied diagnosis. To minimize these cofounding factors, we set the time interval to six months before or after diagnosis for retrieval of analgesics. Despite this time interval there may still be an overlap in diagnoses and treatments that could impact our results, and this is acknowledged as a major limitation of the study. The inclusion of the complete population inherently reduces the impact of potential confounding factors by virtue of the large sample size and their effects are likely to be equally distributed among the study groups due to the comprehensive dataset. In addition, we compared the retrieval of opioids in the total population. The proportion of people retrieving opioids for the diagnosis studied was higher than in the population at large. These results indicate that the studied diagnoses have a major impact on opioid prescription and retrieval in the study population. From the total population data, the age group distribution of opioid retrieval also shows trends of the lowest proportion of patients retrieving opioids in the youngest age group and increasing by age group.

In the diagnosis studied, the age distribution is not expected to be the same for diagnoses such as lumbar spine stenosis; for example, lumbar root canal stenosis heavily depends on degeneration of the spine, which increases with age [18]. If confounding factors significantly influenced our diagnostic groups that retrieved a large amount of opioids, we expect to see the same pattern as we see in the total population; however, we did not see this pattern. Our assessment is again that medications are prescribed specifically for our included diagnoses. A limitation of our study is that a patient with one of our studied diagnoses may also have an additional diagnosis. Thus, patients can be included in multiple subgroups in terms of diagnosis codes. In addition, we were unable to ascertain patient compliance to treatment. That is, being prescribed a medication does not mean that the patients take the medication as prescribed even if they retrieve it at the pharmacy; however, this issue does not affect the results of the current study.

Moreover, our study design allowed for the inclusion of patients who may have transitioned between NG and OG analgesics during the study period, as well as those who received both types concurrently. This closely reflects the real-world clinical practice and enhances the external validity of our findings. However, it is acknowledged that this approach may introduce potential confounding factors and impact the interpretation of the results. However, the large sample size of our study population provides robustness to detect any differences in outcomes between the treatment groups. Any observed differences between NG and OG groups are likely to be clinically meaningful and indicative of real-world treatment patterns and outcomes in patients with neuropathic low back pain.

The data coverage is a major strength of the study as it includes all data from the VEGA and Digitalis registries, which include the total population of the Västra Götaland region. A consequence of the inherent limitation to aggregated non-identifiable data output from the VEGA registry is that patients 0–18 years old, i.e. children were included in the study population. In proportion to all individuals within the studied diagnosis, the children (0–18 years old) accounted for 0.40 − 0.04%. Despite the inclusion of patients < 18 years old the treatment options remain the same for all ages as does the conclusions drawn from our results.

Moreover, the completeness of these registries is total as all diagnoses and prescribed medications in this region are registered. The fact that the guidelines in Sweden for treating neuropathic low back pain have been basically the same since at least 2012 might have made all involved doctors and predominantly primary care physicians aware of the treatment recommendations, which would increase the concordance of prescribed treatment among physicians. Improved treatment of patients with neuropathic low back pain in public health care should improve patient satisfaction and may also lower the cost to both society and personal disability [19].

Earlier studies show that low back pain is more common among women than men [19]; our study shows that women with diagnosed neuropathic low back pain are also more likely to receive neuropathic medication than men. The expression and description of symptoms might differ between the genders and therefore be a contributing factor to this difference. It is very important to correctly diagnose neuropathic back pain. Using pain maps or validated screening questionnaires are easy and accessible objective tool to increase the validity of diagnosis [20, 21].

## Conclusions

The trends identified in this study suggest a general increase in the prescription rate and consequently patients using neuropathic analgesics for neuropathic pain associated with the studied diagnoses. The study also establishes neuropathic medications to be a more common choice of treatment for the studied diagnosis as compared to opioids. However, opioid treatment remains currently more common. The results indicate that there is room for further improvements in the way health care treats neuropathic low back pain.

## Data Availability

The datasets used and/or analyzed during the current study are available from the corresponding author on reasonable request.

## Abbreviations

NSAID: Non steroid anti–inflammatory drug
REK: List–treatment recommendation list
NG: Neuropathic group
OG: Opioid group
